# Supplementary material for: Fermentation Enhanced Biotransformation of Compounds in the Kernel of Chrysophyllum albidum
Source: Molecules. 2020 Dec 19;25(24):6021. doi: 10.3390/molecules25246021 (PMC7768532; doi:10.3390/molecules25246021)
Supplement: Supplementary file 1 [file molecules-25-06021-s001.pdf]

Supplementary Materials for

Fermentation Enhanced Biotransformation of Compounds in the Kernel of *Chrysophyllum albidum*

Table S1. Complete compounds present in the GC-MS analysis of the unfermented aqueous extract of *C. albidum*.

Peaks	tR	Area (%)	Similarity index (%)	Class of Compound	IUPAC Name	Common Name
1	5.06	0.56	90	Alcohol	furan-2-ylmethanol	Furfuryl alcohol
2	5.39	0.33	80	Carboxylic acid	6-oxo-1H-pyrimidine-4-carboxylic acid	6-Hydroxy-4-pyrimidinecarboxylic acid
3	4.48	0.26	80	Alcohol	3-methylcyclopentane-1,2-diol	3-methyl-1,2-cyclopentanediol
4	5.74	1.27	89	Pyrazine	2,5-dimethylpyrazine	2,5-dimethylpyrazine
5	5.81	0.46	77	Ketone	4-Penten-2-one, 3-cyclohexyl-	3-cyclohexylpent-4-en-2-one
6	5.93	0.47	78	Ketone	2-methylcyclopentan-1-one	2-Methylcyclopentanone
7	6.18	0.22	80	Alcohol	5-methylfuran-2-ylmethanol	5-Methylfurfuryl alcohol
8	6.32	0.11	75	Imidazole	1,4,5-trimethylimidazole	1,4,5-Trimethyl-1H-imidazole
9	6.52	0.08	73	Fatty acid	(*E*)-non-2-enoic acid	Nonylenic acid
10	6.77	0.30	71	Carboxylic acid	ethyl 1-(methoxymethyl)imidazole-4-carboxylate	Imidazole-4-carboxylic acid, 1-methoxymethyl-, ethyl ester
11	6.83	0.65	79	Pyrazine	2-ethyl-3-methylpyrazine	2-ethyl-3-methylpyrazine
12	7.05	0.11	78	Ketone	2-hydroxypentan-3-one	2-Hydroxy-3-pentanone
13	7.82	0.93	75	Aldehyde	2,3-dihydroxypropanal	Glyceraldehyde
14	7.88	1.39	65	Alcohol	2-(2-methylpropylsulfanyl)ethanol	2-(Isobutylthio)ethanol
15	9.13	2.90	86	Ketone	3,5-Dihydroxy-6-methyl-2,3-dihydro-4H-pyran-4-one	2,3-Dihydro-3,5-dihydroxy-6-methyl-4h-pyran-4-one
16	9.44	0.74	66	Fatty acid	11-aminoundecanoic acid	11-Aminoundecylic acid
17	9.96	0.65	80	Alcohol	1-(4a,6,7,8a-tetrahydro-4H-[1,3]dioxino[4,5-b][1,4]dioxin-4-yl)ethanol	1,3:2,4-Dimethylene-d-epirhamnitol
18	10.39	0.88	75	Ketone	5-Methoxy-4-pyrimidinol	5-methoxy-4(3H)-pyrimidinone
19	11.25	1.31	91	Indole	1H-indole	Indole
20	11.30	1.09	77	Amine	1-(oxolan-2-yl)-*N*-(oxolan-2-ylmethyl)methanamine	Di(2-Tetrahydrofurylmethyl)amine
21	11.43	0.61	71	Oxazole	5-methyl-3a-nitro-2,3,4,5-tetrahydro-[1,2]oxazolo[2,3-b][1,2]oxazole	2-Methyl-8-nitroisoxazolizidine
22	11.83	0.35	68	Alkane	1-methoxyundecane	Methyl undecyl ether

23	12.34	0.31	73	Alkane	Cyclohexane, 1,2,3-trimethoxy-, stereoisomer	1,2,3-Trimethoxycyclohexane
24	12.39	0.70	76	Indole	3-(piperidin-1-ylmethyl)-1H-indole	*N*-Skatylpiperidine
25	12.64	0.10	70	Amine	*N*-methyl-9-borabicyclo[3.3.1]nonan-9-amine	*N*-methyl-9-borabicyclo[3.3.1]nonan-9-amine
26	13.38	0.92	90	Nitrile	2,4,6-trimethylbenzonitrile	Mesitonitrile
27	13.57	0.38	67	Nitrile	5,12-Naphthacenedione, 10-((3-(3-cyano-4-morpholinyl)-2,3,6-trideoxy-.alpha.-L-lyxo-hexopyranosyl)oxy)-7,8,9,10-tetrahydro-6,8,11-trihydroxy-8-(hydroxyacetyl)-1-methoxy-, (8S-cis)-	Cyanomorpholinoadriamycin
28	13.36	0.24	80	Fatty acid ester	Adenosine, N6-phenylacetic acid	Methyl 12-methylmyristate
29	13.89	0.57	74	Carboxylic acid	2-[4-[[9-[(2R,3R,4S,5R)-3,4-dihydroxy-5-(hydroxymethyl)oxolan-2-yl]purin-6-yl]amino]phenyl]acetic acid	Adenosine, *N*6-phenylacetic acid
30	14.57	24.38	80	Alcohol	2-(hydroxymethyl)-2-nitropropane-1,3-diol	Tris(hydroxymethyl)nitromethane
31	15.39	0.45	72	Alkane	1-ethenylsulfanyloctane	Octane, 1-(ethenylthio)-
32	15.52	5.76	83	Galactoside	.alpha.-D-Galactopyranoside, methyl	Methyl-alpha-D-galactoside
33	15.76	2.21	78	Ketone	3,5-dihydroxy-6-(hydroxymethyl)oxan-2-one	3-Deoxy-d-mannoic lactone
34	15.87	1.44	70	Aldehyde	Methyl-n-nonylacetaldehyde	2-Methylundecanal
35	15.95	1.82	68	Alcohol	2-(1H-indol-3-yl)ethanol	Tryptophol
36	16.04	1.47	63	Fatty acid ester	(*E*)-3-methylhex-2-enoic acid	*trans*-3-Methyl-2-hexenoic acid
37	16.41	0.98	66	Ketone	8a-methyl-1,3,4,4a,5,6,7,8-octahydronaphthalen-2-one	8a-methyloctahydro-2(1H)-Naphthalenone
38	16.61	0.49	82	Pyridine	5H-Indeno[1,2-b]pyridine	4-Azafluorene
39	16.88	1.28	75	Fatty acid ester	hexadecyl(trimethyl)azanium;octadecanoate	Hexadecyltrimethylammonium Stearate
40	17.10	3.36	82	Tryptamines	*N*,*N*-dimethyl-2-[5-(1,2,4-triazol-1-ylmethyl)-1H-indol-3-yl]ethanamine	Rizatriptan
41	17.69	3.01	87	Beta-carboline	9H-Pyrido[3,4-b]indole	Norhaman
42	17.74	3.44	74	Ketone	1-pyrido[3,4-b]indol-9-ylethanone	Norharmane, *N*-acetyl-
43	18.04	4.33	85	Fatty acid ester	Methyl cis-6-octadecenoate	Methyl Petroselinate
44	18.24	6.65	80	Ketone	(1E)-4,4-Dimethyl-1-(4-morpholinyl)-1-penten-3-one	Pent-1-en-3-one, 4,4-dimethyl-1-(4-morpholino)-
45	18.31	6.80	75	Alkane	2,7-Dioxatricyclo[4.4.0.0(3,8)]decane	2,7-Dioxatricyclo[4.4.0.0(3,8)]decane
46	18.41	4.86	76	Ketone	2,4-Dihydroxy-5,6-dimethylpyrimidine	5,6-Dimethyluracil
47	18.73	0.42	55	Carboxylic acid	ethyl 4-ethoxycarbonyloxyazepane-1-carboxylate	1-Hexahydroazepinecarboxylic acid, 4-ethoxycarbonyloxy-, ethyl ester

48	19.02	0.19	60	Alcohol	4-ethenyl-1-ethyl-2-methyl-2,3,4a,5,6,7,8,8a-octahydroquinolin-4-ol	1-Ethyl-2-methyl-4-vinyldecahydro-4-quinolinol
49	19.08	0.20	56	Carboxylic acid	E-3-Tetradecen-1-ol acetate	3E-Tetradecenyl acetate
50	19.16	0.15	59	Ketone	9-Oxabicyclo[3.3.1]nonan-2-one	5-Hydroxy-9-oxabicyclo[3.3.1]nonan-2-one
51	19.22	0.08	59	Alcohol	9-Oxabicyclo[3.3.1]nonane-2,6-diol	9-Oxabicyclo[3.3.1]nonane-2,6-diol, (1R,2R,5R,6R)- (9CI)
52	19.36	0.19	61	Fatty acid ester	9,12,15-Octadecatrienoic acid, 2-[(trimethylsilyl)oxy]-1-[[(trimethylsilyl)oxy]methyl]ethyl ester, (Z,Z,Z)-	2-Monolinolenin, 2TMS derivative
53	19.44	1.75	68	Indole	2,3-dimethyl-4-azatricyclo[5.3.1.04,11]undeca-1(10),2,7(11),8-tetraene	1,7-Dimethylene-2,3-dimethylindole
54	19.51	1.77	82	Indole	6-methyl-2-(3-pyridinyl)-1H-indole	6-methyl-2-(3-pyridyl)indole
55	19.58	0.33	69	Fatty acid ester	1-cyclopentylethyl 4-cyanobenzoate	1-cyclopentylethyl 4-cyanobenzoate
56	19.66	0.63	59	Alkane	1-methyl-1-octoxysiletane	1-Methyl-1-n-octyloxy-1-silacyclobutane
57	19.85	0.76	73	Amino acid	2-amino-3-(1H-indol-3-yl)propanoic acid	DL-Tryptophan
58	19.92	0.43	62	Ketone	11H-isoindolo[2,1-a]quinazolin-5-one	isoindolo[2,1-a]quinazolin-5(11H)-one
59	20.18	0.35	64	Fatty acid ester	9,12,15-Octadecatrienoic acid, 2-[(trimethylsilyl)oxy]-1-[[(trimethylsilyl)oxy]methyl]ethyl ester, (Z,Z,Z)-	2-Monolinolenin, 2TMS derivative
60	20.28	0.29	56	Indole alkaloid	(9R,21S)-2,12-diazahexacyclo[14.2.2.19,12.01,9.03,8.016,21]henicosa-3,5,7-triene	Aspidofractinine
61	20.81	0.17	52	Fatty acid ester	(2,6-ditert-butyl-4-methoxyphenyl) 4-cyano-2-nitrobutanoate	Butanoic acid, 4-cyano-2-nitro-, 2,6-bis(1,1-dimethylethyl)-4-methoxyphenyl ester
62	21.78	0.65	71	Fatty acid ester	2,3-dihydroxypropyl (Z)-octadec-9-enoate	Glyceryl monooleate

Table S2. Complete compounds present in the GC-MS analysis of the aqueous extract of fermented *C. albidum.*

Peaks	tR	Area (%)	Similarity index (%)	Class of Compound	IUPAC Name	Common Name
1	5.58	0.92	87	Pyrazine	2,5-dimethyl pyrazine	2,5-dimethyl pyrazine
2	6.85	0.66	82	Alcohol	3-Bromopentan-2-ol	Theo-3-Bromo-2-pentanol
3	6.85	28.57	94	Carboxylic acid	(2S)-2-hydroxypropanoic acid	L-Lactic acid
4	8.76	4.07	80	Akylglycerol	3-(2,3-dihydroxypropoxy)propane-1,2-diol	Diglycerol
5	9.16	4.66	79	Ketone	3,5-Dihydroxy-6-methyl-2,3-dihydro-4H-pyran-4-one	2,3-Dihydro-3,5-dihydroxy-6-methyl-4h-pyran-4-one
6	11.21	1.51	92	Indole	1H-indole	Indole
7	12.39	0.80	79	Indole	6-methyl-1H-indole	6-Methylindole
8	12.88	0.25	65	Ketone	oxepane-2,7-dione	Adipic anhydride
9	13.36	0.51	86	Nitrile	2,4,6-trimethylbenzonitrile	Mesitonitrile
10	13.69	0.86	67	Alcohol	Bicyclo[2.2.1]heptane-2,3-diol, 1,7,7-trimethyl-, (exo,exo)-	Camphanediol
11	14.12	1.98	74	Diterpene glycoside	[(2S,3R,4S,5S,6R)-3,4,5-trihydroxy-6-(hydroxymethyl)oxan-2-yl] (1R,4S,5R,9S,10R,13S)-13-[(2S,3R,4S,5S,6R)-4,5-dihydroxy-6-(hydroxymethyl)-3-[(2S,3R,4S,5S,6R)-3,4,5-trihydroxy-6-(hydroxymethyl)oxan-2-yl]oxyoxan-2-yl]oxy-5,9-dimethyl-14-methylidenetetracyclo[11.2.1.01,10.04,9]hexadecane-5-carboxylate	Stevioside
12	14.39	0.84	61	Fatty acid esters	dibutan-2-yl octanedioate	Octanedioic acid, bis(1-methylpropyl) ester
13	15.58	0.48	72	Amine	1-dicyclohexylphosphanyl-*N*,*N*-dimethylmethanamine	Methanamine, 1-(dicyclohexylphosphino)-N,N-dimethyl-
14	16.06	1.78	65	Alcohol	4,4-Dimethyl-cyclohex-2-en-1-ol	2-Cyclohexen-1-ol, 4,4-dimethyl-
15	16.62	0.99	82	Pyridine	5H-indeno[1,2-b]pyridine	4-Azafluorene
16	16.90	0.78	77	Amine	5-(1H-indol-3-yl)-2-methylpentan-2-amine	3-(4,4-Dimethyl-4-aminobutyl)-indole
17	17.12	1.91	75	Tryptamines	N,N-dimethyl-2-[5-(1,2,4-triazol-1-ylmethyl)-1H-indol-3-yl]ethanamine	Rizatriptan
18	17.31	4.46	72	Indole	1H-Pyrido[3,4-b]indole, 2,3,4,9-tetrahydro-1-methyl-	Tetrahydroharman Methtryptoline dl-eleagnin
19	17.56	6.90	72	Indole alkaloid	1-methyl-9H-pyrido[3,4-b]indole	Harman
20	17.71	5.07	95	Beta-carboline	9H-Pyrido[3,4-b]indole	Norhaman
21	18.00	0.23	84	Fatty acid esters	methyl 8-[2-[[2-[(2-ethylcyclopropyl)methyl]cyclopropyl]methyl]cyclopropyl]octanoate	Methyl 8-[2-((2-[(2-ethylcyclopropyl)methyl]cyclopropyl)methyl)cyclopropyl]octanoate

22	18.04	1.73	85	Fatty acid esters	7-Hexadecenoic acid, methyl ester, (Z)-	formyl 7E-hexadecenoate
23	18.19	4.89	77	Ketone	(E)-4,4-dimethyl-1-morpholin-4-ylpent-1-en-3-one	Pent-1-en-3-one, 4,4-dimethyl-1-(4-morpholino)-
24	18.34	9.38	82	Alkane	2,5-methano-2H-furo[3,2-b]pyran, hexahydro-	2,7-dioxaisotwistane
25	18.46	1.80	73	Fatty acid	Eicosanoic acid	Arachidic acid
26	18.74	2.83	55	Ketone	1,3-dimethyl-5-(oxiran-2-ylmethyl)-5-propan-2-yl-1,3-diazinane-2,4,6-trione	2,4,6(1H,3H,5H)-Pyrimidinetrione, 1,3-dimethyl-5-(1-methylethyl)-5-(oxiranylmethyl)-
27	19.02	2.24	56	Alcohol	6-decylsulfonylhexane-1,2,3,4,5-pentol	d-Mannitol, 1-decylsulfonyl-
28	19.44	0.71	59	Ketone	3-sulfanylidene-1,2,4-triazaspiro[5.5]undecan-5-one	
29	19.51	2.15		Indole	6-methyl-2-pyridin-3-yl-1H-indole	6-methyl-2-(3-pyridyl)indole
30	19.66	1.37	57	Alkane	1-methyl-1-octoxysiletane	1-Methyl-1-n-octyloxy-1-silacyclobutane
31	19.85	1.37	68	Ketone	2-Allyl-2-(1H-indol-3-ylmethyl)-malonic acid	Cytosine riboside, TMS
32	19.93	0.51	48	Fatty acid esters	ethyl 3-(3-ethoxy-3-oxopropyl)sulfanylpropanoate	Diethyl thiodipropionate
33	20.19	0.34	56	Alkyne	1-chloroheptadec-4-yne	1-Chloro-4-heptadecyne
34	20.29	1.68	71	Alkaloid	N-[2-(1H-indol-3-yl)ethyl]-N-methylacetamide	Nb-Acetyl-Nb-methyltryptamine
35	20.33	0.30	55	Silane	[(3aS,4S,6R,7S,7aR)-2-butyl-6-methoxy-4-methyl-4,6,7,7a-tetrahydro-3aH-[1,3,2]dioxaborolo[4,5-c]pyran-7-yl]oxy-trimethylsilane	alpha.-l-Galactopyranoside, methyl 6-deoxy-2-*O*-(trimethylsilyl)-, cyclic butylboronate
36	20.58	0.09	52	Carboxylic acid	[(3S,8S,9S,10S,13S,14S)-10-(hydroxymethyl)-13-methyl-2,3,4,7,8,9,11,12,14,15,16,17-dodecahydro-1H-cyclopenta[a]phenanthren-3-yl] acetate	Androst-5-ene-3,19-diol, 3-acetate, (3.beta.)-
37	20.81	0.12	53	Silane	ethoxy-dimethyl-[(E)-3-phenylprop-2-enoxy]silane	Silane, dimethyl(3-phenylprop-2-enyloxy)ethoxy-
38	20.90	0.07	58	Fatty acid esters	9,12,15-Octadecatrienoic acid, 2-[(trimethylsilyl)oxy]-1-[[(trimethylsilyl)oxy]methyl]ethyl ester, (Z,Z,Z)-	2-Monolinolenin, 2TMS derivative
39	21.03	0.21	50	Amide	*N*-(2-amino-2-oxoethyl)tetradec-2-ynamide	2-Myristynoyl-glycinamide

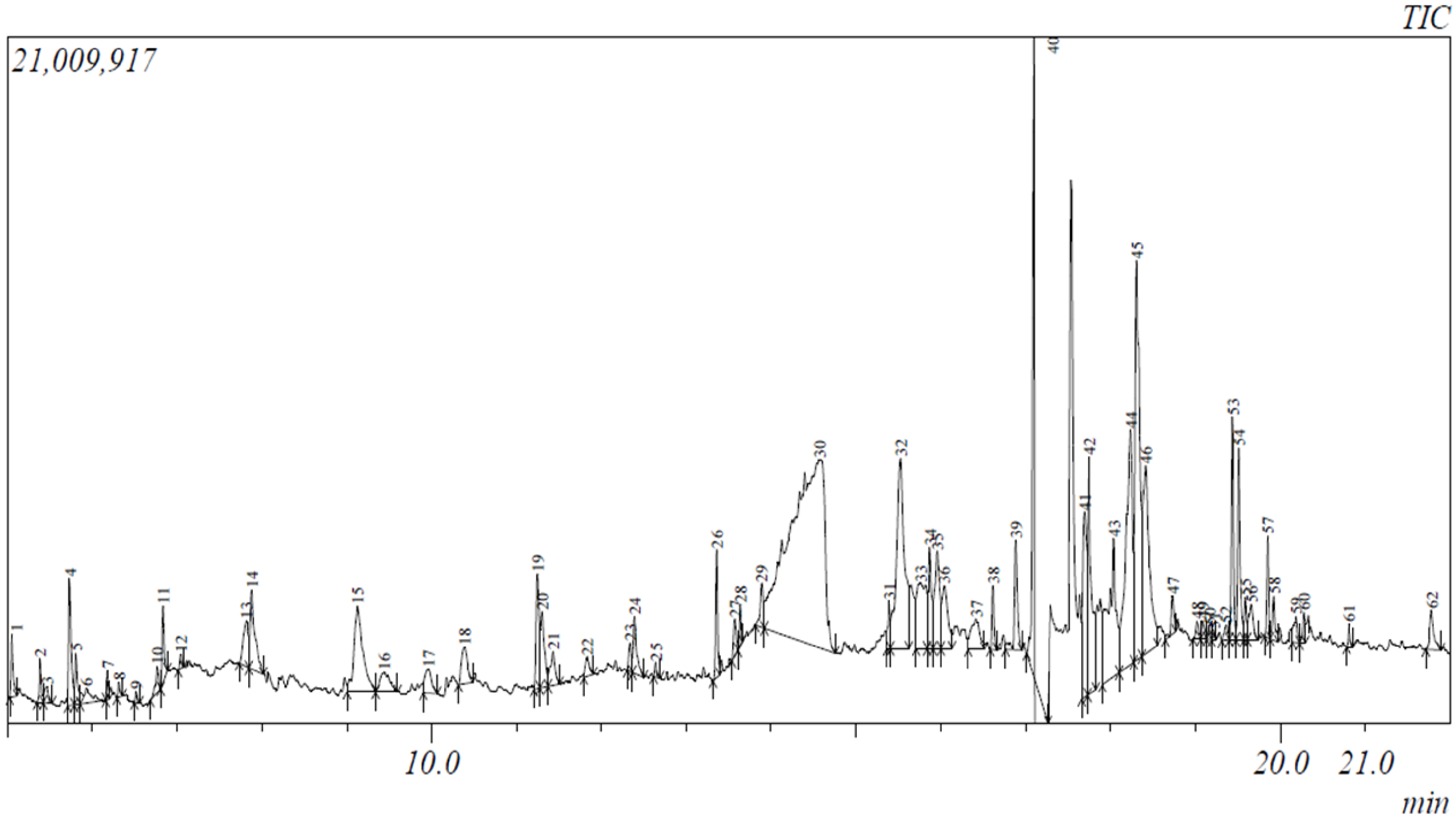

Figure S1. Chromatogram generated during analysis of extract from unfermented C *albidum*.

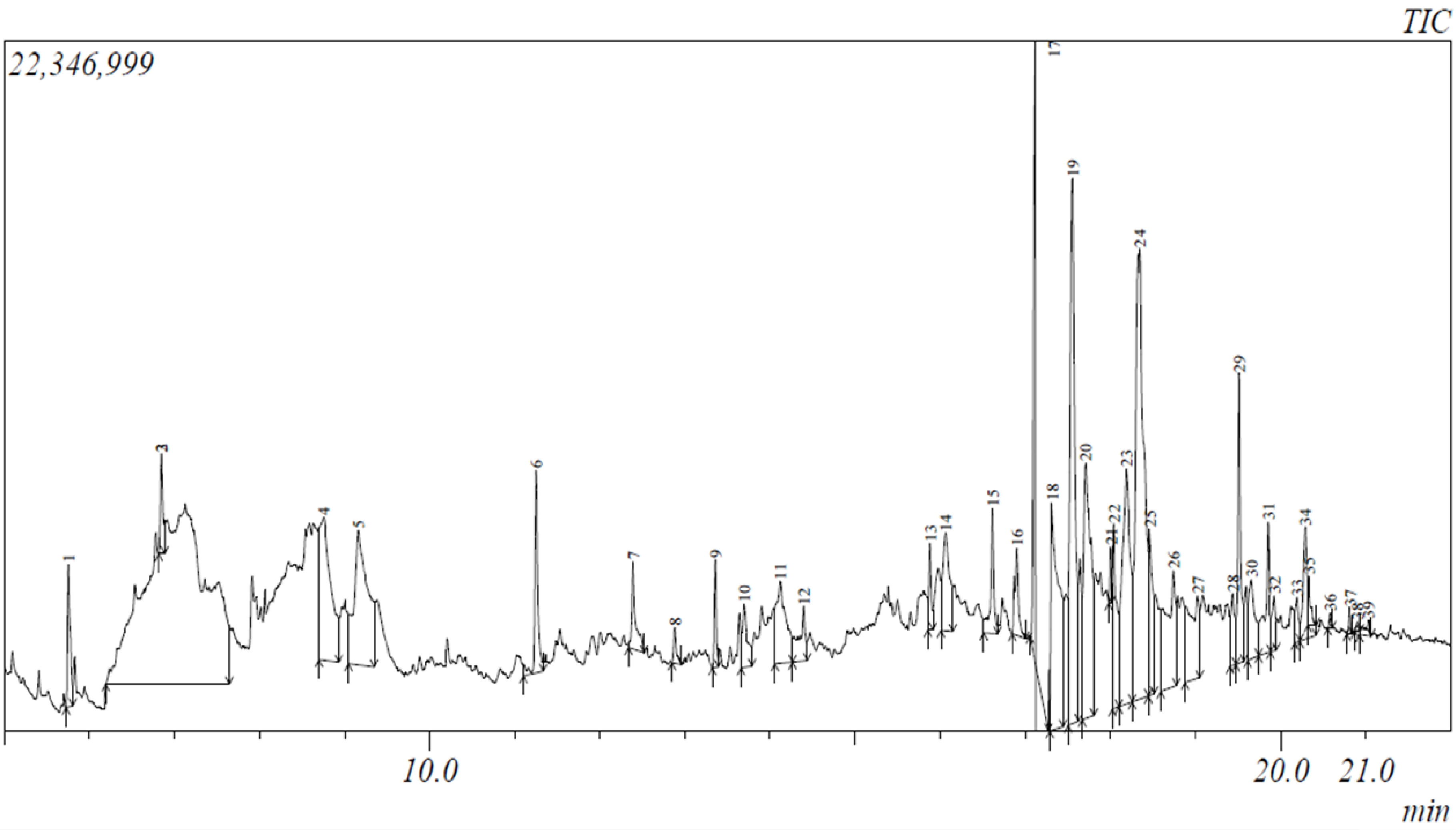

Figure S2. Chromatogram generated during analysis of extract from fermented C *albidum*.
